# Cross Sectional Analysis of Eurasian Skull Anatomy for 3D Cephalometry—Normative Data Reveal Four Different Skull Types

**DOI:** 10.3390/jpm13061018

**Published:** 2023-06-19

**Authors:** Leon Ludwigs, Christian Pape, Helena Sophie Visse, Christoph Runte, Ulrich Meyer, Dieter Dirksen

**Affiliations:** 1 Department of Prosthetic Dentistry and Biomaterials, University of Münster, Albert-Schweitzer-Campus 1, Building W30, D-48149 Münster, Germany; 2 Clinic for Jaw, Face and Skull-Surgery Münster, Schorlemerstraße 26, D-48143 Münster, Germany

**Keywords:** 3D cephalometry, norm skull, skull anatomy, Procrustes analysis

## Abstract

The unsolved problem in three-dimensional surgical planning for patients with facial deformity, dysgnathia, or asymmetry is the lack of a normative database of “norm skulls” that can be used as treatment objectives. A study was conducted on 90 Eurasian persons (46 male and 44 female adults) for whom cone beam-computed tomography images were available. Inclusion criteria were adult patients with a skeletal Class I pattern, proper interincisal relationship with normal occlusion, the absence of an open bite both in the anterior and posterior region, and a normal and balanced facial appearance; patients with dysgnathia and malformations were excluded. A total of 18 landmarks were digitized and 3D cephalometric measurements were performed and analyzed by means of proportions calculated from the landmarks. Male and female skulls were analyzed, as well as subdivisions revealed by cluster analysis. The data showed that four subtypes of skulls were distinguishable with statistical significance (*p* < 0.05). A male and a female type subdivided in a brachiocephalic and dolichocephalic phenotype could be identified. For each type, a mean shape was calculated by a Procrustes transformation, which, in turn, was used to create four template skulls from a male and a female skull. This was accomplished by fitting the polygon models of the two skulls to each of the two subtypes based on the landmarks marked on them using a thin plate spline transformation. The normative data of the subtypes can individually serve as a guide for orthodontic surgery in the Eurasian population, which is especially helpful in 3D planning and the execution of craniofacial operations.

## 1. Introduction

Three-dimensional technologies now form the basis of a significant expansion of diagnostic and treatment options in dentistry and oral surgery. These include devices such as the intraoral scanner, imaging techniques such as computed tomography (CT), cone beam-computed tomography (CBCT) and magnetic resonance imaging (MRI), as well as the corresponding treatment planning software and CAD/CAM systems. They pave the way for clinicians to significantly improve patient care while reducing treatment planning time [1,2]. These technologies allow the precise three-dimensional reproduction of anatomical structures. However, conventional orthognathic surgery planning is commonly done on computer-assisted two-dimensional surgical simulation systems, which rely on photographs and cephalograms [3,4]. Additionally, a facebow and bite records are used in order to adequately register the patients’ bite and jaw position in an articulator. This way, surgical displacements can be simulated using the patients’ cast models, as this is required for surgical splint manufacturing. Additionally, the lateral cephalometric X-ray is still used for planning orthognathic surgery, although this only provides a two-dimensional image and is not true to scale. While these methods are well established, the use of a mechanical articulator and two-dimensional imaging for planning three-dimensional procedures can lead to imprecisions. Planning and execution in orthognathic surgery is currently shifting from 2D to 3D, as more planning is done digitally and focuses on the jaw. In recent decades, however, computed tomography (CT), especially by cone-beam CT (CBCT), has become the gold standard for pre- and postoperative assessments, and CT superimposition is state-of-the-art when it comes to orthognathic surgical planning and evaluation. Positioning is done by moving jaws in all directions according to pitch, roll and jaw parameters, and the jaw needs to be placed correctly in the 3D space [5,6]. Swennen et al. introduced three-dimensional (3D) cephalometry with the 3D anatomic cartesian reference system, making the bridge between 2D and 3D assessments [7]. Geometrical distortions in craniofacial malformations, craniofacial asymmetries, structural overlap and incorrect positioning of the head can affect the accuracy of the two-dimensional assessment [8,9].

Gateno et al. [10], with their model of analysis, maintained what is positive in 2D cephalometry but attempted to overcome its limits. In their report, the authors presented a new three-dimensional cephalometry to compensate for the unreliability of internal reference systems, and render three-dimensional measurements more accurate, allowing for the lack of tools to assess and measure complex 3D anatomy. Today, in orthodontics and orofacial orthopedics, the cephalometric 3D analysis is an important tool. For some of the above problems in two-dimensional design, three-dimensional simulation systems using CBCT data have proven to be a solution. Such 3D cephalometry allows for a more detailed analysis of the craniofacial structure [11]. With this approach, it is possible not only to detect more easily but also to quantify craniofacial malformations, asymmetries, longitudinal growth and small occlusal changes. The 3D images have been shown to more accurately capture anatomical information and provide more precise quantitative measurements compared to 2D images [12]. Since most common cephalometric measurements have been shown to be compatible with 3D volumetric images, there is an effort to obtain a standardized reference for the craniofacial structure of normal dentofacial patterns in a population. In a 3D approach, craniofacial and maxillofacial segments can be defined and positioned by translation with respect to the three spatial axes (x, y and z), and adjustments are made by rotation about these axes, representing “roll”, “pitch” and “yaw” [13,14]. Whereas the technique of segmentation and movement is technically solved and can be easily performed, the unsolved problem in three-dimensional planning is the lack of a normative database of “norm skulls”, as these skulls can be used as the positioning data of jaw movements. Until now, digital surgical planning in terms of a definitive jaw position has been based more on “eye measurement”. Normative values of 3D cephalometry were obtained for different ethnic groups. Different facial characteristics and average values were found between ethnic groups, which should be taken into account in treatment planning [15,16,17,18,19]. 

Due to recent advances in image processing techniques and the need for accurate craniofacial analysis, a three-dimensional (3D) approach to the cephalo-metric landmarks obtaining 3D computerized tomography (CT) images is gaining preference over the conventional 2D techniques [20,21,22,23]. Personalized medicine tends to incorporate intrinsic features in the planning of therapy. The question arises whether the overlay of norm skulls can be applicated more precisely to the phenotypic pattern of individual skulls. No one to date has analyzed whether skulls with a normal and balanced facial appearance, skeletal Class I pattern and a proper interincisal relationship with normal occlusion can be subdivided and clustered in groups, so we aimed to investigate 3D landmark positions biostatistically. That is, we wanted to investigate whether an analysis of skull shape based on a limited number of characteristic points would reveal statistically significant differentiable shapes. Our study was conducted with the aim of possibly identifying anatomical variations in a clinical attempt to evaluate treatment efficacy or enhance surgical planning accuracy in orthognathic and malformation surgery.

## 2. Materials and Methods

Anonymized cone beam-computed tomography (CBCT) of physiological human skulls of 90 Eurasian persons (46 male and 44 female adults) were used for the study. Inclusion criteria were skeletal angle class I pattern, proper interincisal relationship with normal occlusion, absence of an open bite both in the anterior and posterior region and a normal and balanced facial appearance. The study was conducted on all patients fulfilling the inclusion criteria from 2020–2022. The inclusion criteria were determined after a thorough clinical investigation. In addition, the Medical Faculty of the University of Münster provided one male and one female skull, which were scanned with a computed tomography (CT) in the highest resolution, in order to provide a template skull for further investigation in our study. The following parameters were used: CBCT: 576 × 576 pixel, pixel spacing 0.4 × 0.4 mm, slice thickness 0.4 mm; CT: 512 × 512 pixel, pixel spacing 0.36 x 0.36 mm; slice thickness 0.4 mm. 

DICOM data were saved and subsequently imported into the open-source software 3D Slicer (version 4.11.2021, www.slicer.org (accessed on 14 June 2023)). In order to create a virtual model of the facial skull, automatic threshold-based segmentation was performed. Artifacts triggered by prosthetic and conservative restorations were manually corrected in affected slices by using the tools “scissor” and “erase” in coronal view. Pseudo foramina were closed using the “paint” tool. The mesh was exported as an STL file and imported into MeshLab open-source software (version 2022.02, www.meshlab.net (accessed on 14 June 2023)). The remaining artifacts of the created models were removed. The created data sets were exported to PLY format.

**Figure 1:**
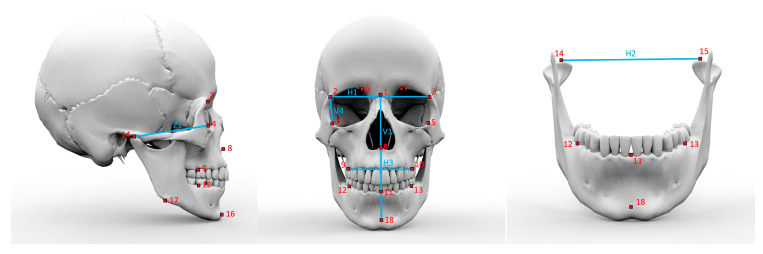
Landmarks and distances used for classification of the skull as described in Table 1 and Table 2.

Afterwards, 18 different landmarks (Table 1, Figure 1) were tagged on each skull by the same person. The distances between the landmarks (Table 2) and skeletal proportions (Table 3) were defined to analyze skeletal phenotype patterns. 

### 2.1. Evaluating Sexual Dimorphism

To analyze the gender-specific characteristics of the skulls, shapes were first defined on the basis of the recorded landmarks. For this purpose, the landmarks were arranged in an arbitrary but fixed order according to Table 1. Next, they were registered, i.e., oriented and superimposed in a common coordinate system by a best-fit algorithm. This was achieved by means of a Procrustes transformation through translation, rotation and scaling [23]. During the Procrustes analysis, a mean shape was also calculated for both sexes. Furthermore, the proportions listed in Table 3 were determined for male and female skulls, respectively. To explore whether there are significant gender-specific differences in skeletal characteristics, the individual proportions of both groups were compared. After a test for normal distribution (Kolmogorov–Smirnov), this was done by *t*-tests. The *p*-values were adjusted by a Benjamini–Hochberg correction for multiple tests. 

For the statistical evaluation of the collected data, as well as for all programming tasks, the statistic software and programming language “R” (version 4.2.2, www.r-project.org (accessed on 14 June 2023)) was used. Procrustes analysis was carried out with the “R”-package “shapes”.

### 2.2. Identifying Sub-Phenotypes within the Sex-Specific Groups

For both sexes, a cluster analysis was performed. This machine learning approach groups similar skulls based on their characteristics and provides insight into potential subgroups within the population. Here, the method was used to identify potential sub-phenotypes within female and male skulls based on their proportions. This task was carried out by k-means-clustering with the “R”-package “factoextra”. The number of clusters has to be defined in advance and was set to two, both for practical consideration and in accordance with the outcome of the elbow-criterion for determining the optimal number of classes. The proportions for the sub-group of each sex were compared using pairwise *t*-test to determine if there are significant differences in skeletal characteristics between sub-phenotypes.

### 2.3. Creating Template Skulls

To create the template skull, mean shapes were calculated for each of the four groups identified in the previous analyses using generalized Procrustes analysis. Thus, two shape templates were calculated for both male and female skulls. One female and one male skull each were taken from the collection of the Medical Faculty of the University of Münster as source material for the standard skulls to be created. These skulls were scanned using computed tomography, and the images were segmented and converted into polygon models using Mimics (version 23, Materialise NV, Leuven, Belgium) and 3D Slicer software. Finally, for each of the two models, two morphed versions were created with the thin plate spline method (“R” package “Morpho”) by mapping the landmarks identified on them to the landmarks of the four mean-value shapes. This technique allows for a smooth deformation of e.g., 3D polygon objects by interpolation along control points [24] which are represented here by the landmarks. In the present case, this causes the male and female template skulls to adopt the proportions of one of the two sub-phenotypes.

## 3. Results

**Figure 2:**
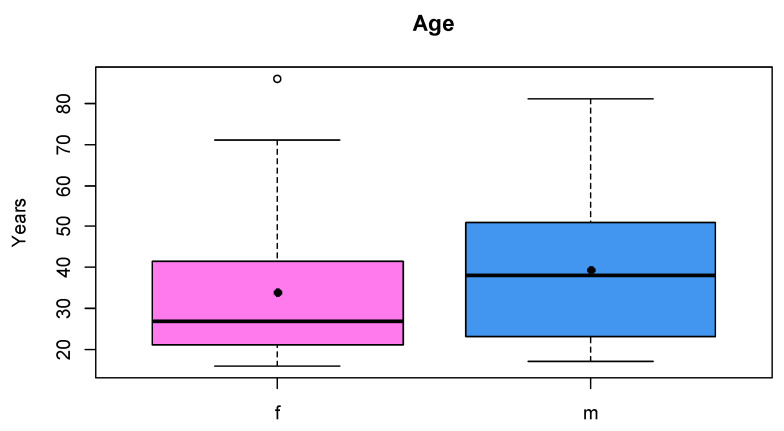
Age distribution of female (f) and male (m) test persons. Black dot denotes the mean value.

**Figure 3:**
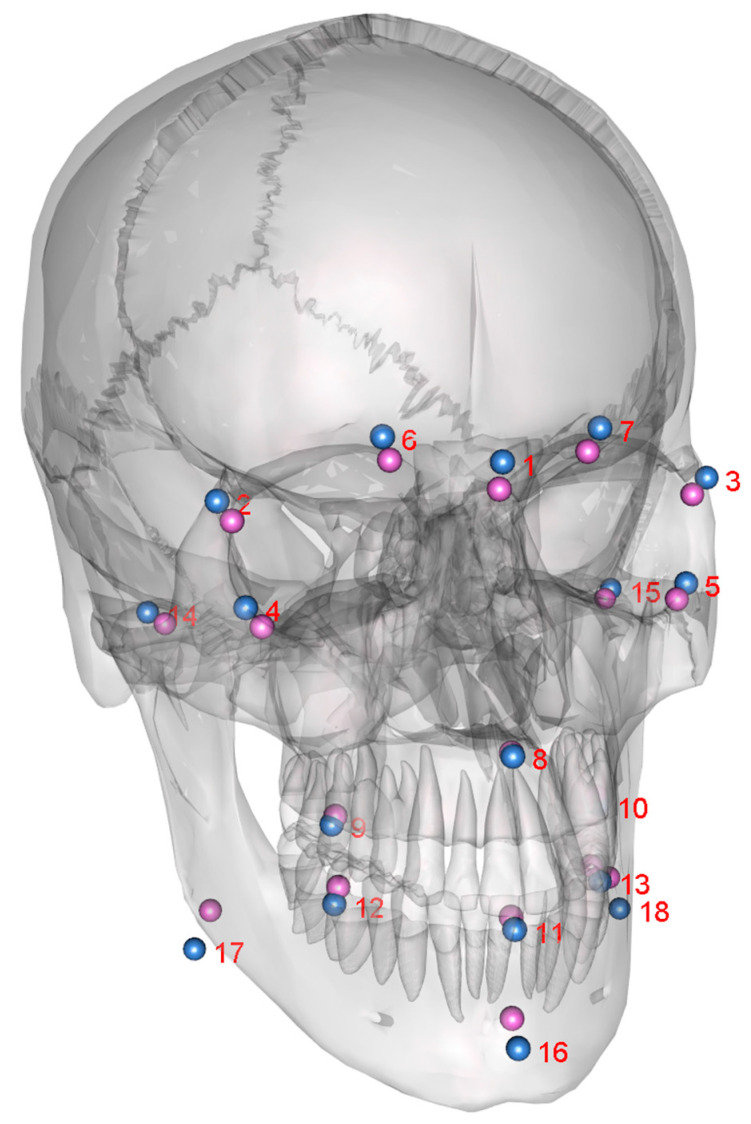
Mean shapes calculated from female (purple) and male (blue) landmarks (as described in Table 1) attached to a skull model for illustration.

**Figure 4:**
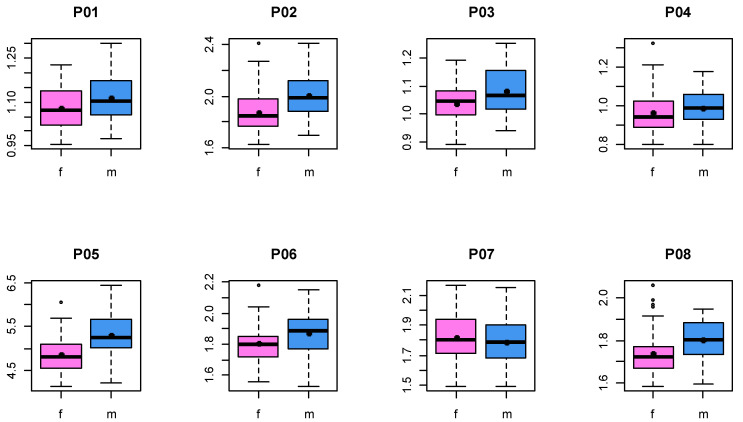
Female (f) and male (m) proportions calculated from shapes (landmarks 1–18). Black dots denote the mean value.

Figure 2 shows the age distributions of the male (mean: 39.4 y) and female (mean: 33.9 y) test persons. For each person, a shape consisting of the 18 landmarks defined in Table 3 was determined. In Figure 3, the mean shapes for both sexes, as revealed by a Procrustes transformation, are displayed. The distributions of the individual proportions (Table 3) were compared for male and female skulls (Figure 4). Kolmogorov–Smirnov tests showed that all distributions could be assumed normal. Subsequently, male and female proportions were compared using *t*-tests. The results (Table 4) showed that, out of the eight investigated proportion, five were significantly different.

**Figure 5:**
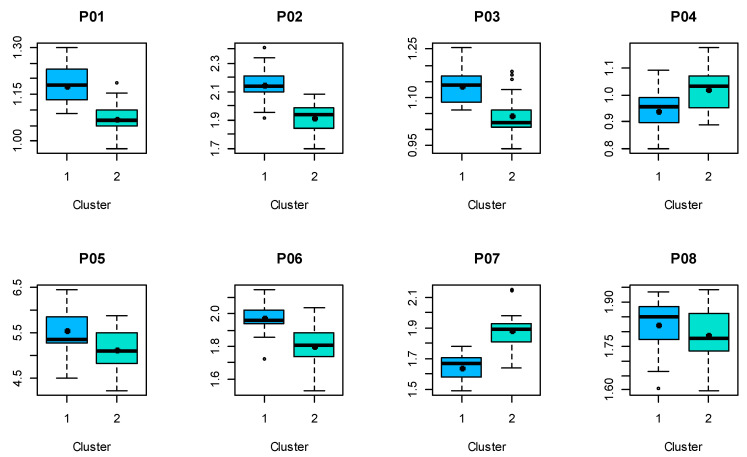
Male proportions calculated from shapes (landmarks 1–18) divided into two clusters. Black dot denotes the mean value.

**Figure 6:**
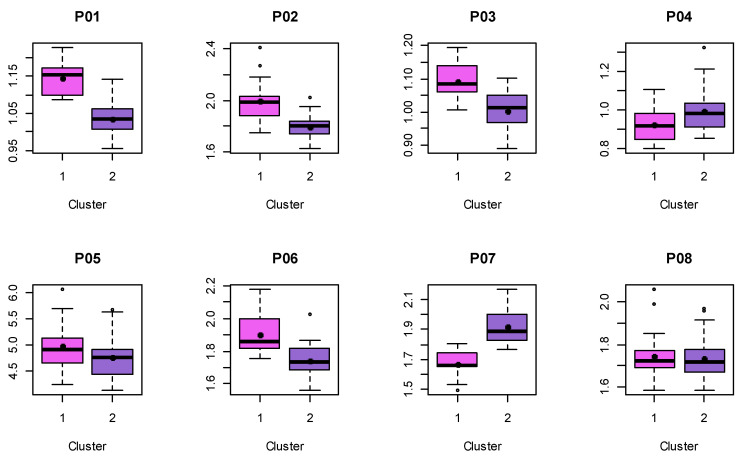
Female proportions calculated from shapes (landmarks 1–18) divided into two clusters. Black dot denotes the mean values.

Next, for each sex, a “k-means” cluster analysis of the respective proportions with two clusters was carried out. The statistical comparisons of these clusters are shown in Figure 5 and Figure 6 for the male and female proportions, respectively. The numerical results of the *t*-tests are listed in Table 5 (male) and Table 6 (female), revealing that, in case of male shapes, seven out of eight proportions differ significantly, while this is the case for six out of eight in case of female shapes.

**Figure 7:**
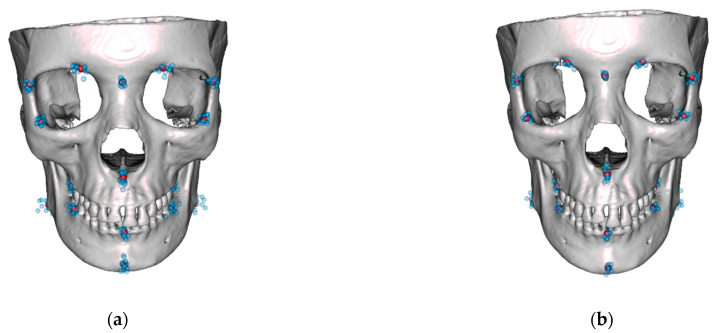
Male template skull morphed with the mean shape of cluster 1 of male shapes (**a**) and cluster 2 of male shapes (**b**). Blue dots denote the landmarks of the respective cluster, purple dots their mean values.

**Figure 8:**
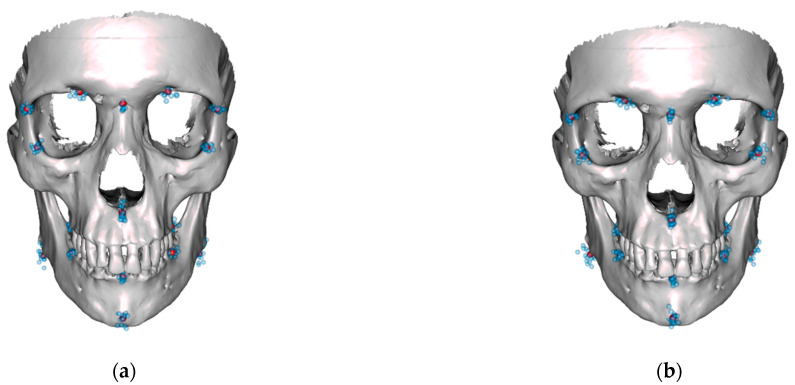
Female template skull morphed with the mean shape of cluster 1 of female shapes (**a**) and cluster 2 of female shapes (**b**). Blue dots denote the landmarks of the respective cluster, purple dots their mean values.

Based on these results, four normative skulls were constructed. For this purpose, a male and a female template skull were morphed to fit the mean shapes determined for each cluster. The results are displayed in Figure 7 and Figure 8.

## 4. Discussion

This study aimed to evaluate phenotypical variance in the adult Eurasian population. A special objective of our study was to analyze whether skulls can be distinguished according to parameters like gender or growth pattern subtypes. An overlay of one unaffected norm skull to a diseased one is currently in use to define the final jaw treatment position, so we aimed to improve the overlay strategy by having more individualized facial phenotypes. The present study defines four different norm skulls in the Eurasian population. These norm skulls can be used as templates in orthognathic surgery planning. They define the treatment objectives in surgical planning in Eurasian patients. Landmarks of CBCTs of dysgnathic patients can be implemented in a planning system, and their gender and phenotype makes it possible to match these patients with one of the four norm skulls.

Dental and maxillofacial patient evaluation and treatment planning is based on an individual dental, skeletal and soft tissue assessment. The implementation of new 3D techniques opened new possibilities in craniofacial research and clinics, as the 3D space mirrors the patients’ situation much more realistically then the 2D environment. CT and CBCT scans are important new tools in dentomaxillofacial diagnostics [25]. The advances in imaging and the elaboration of new software systems have made 3D facial model reconstruction and 3D cephalometric measurement possible, creating a more precise database. The alternative—conventional 2D cephalometric or anthropometric analysis—is limited in all asymmetric patient situations. Different studies show that linear and angular measurements of 3D image models are accurate and reliable when compared with 2D cephalometric analysis [26,27]. Therefore, 3D technologies have become the modern method for evaluation of morphology or deformity. 

Three-dimensional cephalometric standards are different in specific ethnic groups. Additionally, there is a range of variability in the normal anatomy of the craniofacial region. Some landmarks on the 2D and 3D system can be defined as similar points (especially in the lateral view), but the identification of the points in the 3D environment is more extensive. We defined and digitized all hard and soft tissue landmarks according to the definition of skeletal and Farkas for soft tissue by Swennen et al. [14,28]. These landmarks (Table 1) can be reliably set on skeletal models in 3D CBCT or CT scans. They can be easily defined in the most appropriate planar CT or CBCT slice in the axial, coronal and sagittal views. Accurate landmark identification was achieved both for external (e.g., porion, gonion.) and internal (e.g., sella, basion) surface points. Some internal points are more complex to identify (e.g., the sella point), so the views of axial, coronal and sagittal images were used. However, a superimposition of a template skull with an actual case is most meaningful with regard to the nearest surrounding surfaces of the landmarks. Details on surfaces distant from them should be assessed with restraint.

One of the most commonly used 3D cephalometric analyses is based on the work of Gateno et al. [10]. This analysis included six different sections and parameters that are all relevant in the surgical planning of jaw movements (symmetry, transverse, vertical, pitch, anteroposterior and shape). This concept of 3D cephalometric analysis was the basis of the presented work. We refined the reference system to evaluate the cranial skeleton in our study. This form of landmark definition and cephalometric analysis is in broad use, so the data in our study are valid for research and clinical application in a Eurasian population.

The statistical significance of the mean shape differences between male and female subjects in this study was a special finding. While a number of studies reported a marked sexual dimorphism in size and shape [10,29,30,31], no study up to now has shown that the phenotypical differences are statistically significant.

Our analysis revealed significant differences in most parameters between the genders. Studies from other authors in various ethnic groups confirm our findings [16,17,18,19]. Gender variances in our study were present mainly in linear measurement. We found that some parameters (skeletal and soft tissue vertical height), as well as the upper and lower lip length, were larger in our male samples. Our study confirms the findings of 3D phenotype norms in Hong Kong, as well as in the Korean and Turkish population [14,16,19]. The findings that wider sagittal and transversal midfacial parameters were similar among Chinese in Hong Kong, Beijing and Korea were also seen in our study, contrasting findings from the North Karnataka population [32]. Males in our study had a more prominent midface, assessed by the coronal plane through sella turcica, a phenotypical characteristic that was also presented by Cheung et al. [16]. Concerning the lower facial region, a significantly longer mandibular ramus, mandibular body length and a wider gonial width were seen in our male subjects, matching the results of other cephalometric norm studies. 

In addition to the gender-related differences, we found also significant differences in subgroups of the male and female Procrustes mean shapes. Our cluster analysis retained four biologically interpretable components. These are based on the orthodontically well-known types of growth pattern (dolichofacial vs. brachyfacial) and the sagittal expression of phenotype (maxillary and mandibular prognathism or mandibular retrognathism). 

From a developmental, biological and clinical (surgical) point of view, different craniofacial components can be separated: the skull, the midface (with the skull base separating both) and the mandible [33]. With the finding of only four skeletal norm phenotypes (two in each gender group) it becomes easier to plan craniofacial and orthognathic surgeries. Whereas separation (segmentation) of facial bones is an individual decision, the treatment goal can be defined through placement of bones in the individual norm skull phenotype.

There are other classification systems that divide phenotypes into three classes, e.g., based on the cephalic index. This index was introduced as a method of roughly typifying different head shapes with more or less arbitrary subdivisions within a continuous range of values that is presumed to have a normal distribution (see, e.g., [34]). It is based only on one proportion, as it captures the ratio of skull width to skull length in the neurocranial region. The significance of this classification has long been questioned. In a study by Muralidhar et al. [35], correlations between the cephalic index, the facial index and the interincisal distance were found. In this respect, a correlation between the cephalic index and our clusters could be investigated in a further study. In a recent study it was indicated that a correlation between volumetric proportions of four defined levels of the face and attractiveness might exist [36]. It could therefore be a reasonable option to check treatment planning based on norm skulls by predicting its volumetric effects.

Our normative skulls are based on a large number of measurement points, in contrast to simple indices such as the cephalic index and the facial index. Here, eight proportions derived from 18 landmarks are used including the viscerocranium. Therefore, it should be easier to fit them to a patient skull using, for example, a Procrustes transformation. The development of a suitable approach to adapt the template to the current patient situation is the subject of an ongoing research project. One possibility under investigation is superposition based on landmarks in regions least affected by planned interventions by means of a Procrustes transformation. In contrast to other studies examining landmarks from CBCT’s [37,38], based on our cluster analysis, more precise therapy matching according to membership in one of the subgroups may be possible.

The findings of our study must be interpreted in light of the limitation of our study (age and ethnicity). Different populations and ethnic groups have different facial features and averages, which should be considered in the treatment planning. Limitations of the study are found in the inclusion criteria: no malformed patients were chosen as a distinct control group, no multi-center data were acquired and longitudinal observation data were not captioned, so growth development over time could not be analyzed. 

Future areas of research should aim to investigate age-related growth characteristics and the extent of skull deformation in various pathologies (dysgnathic patients and patients with malformative syndromes including craniosynostosis, orofacial clefts and branchial arch diseases).

## 5. Conclusions

This is the first determination and statistical evaluation of 3D cephalometric norms based on CBCT in a Eurasian population. We found that only four norm skulls are statistically different. Facial appearance is largely determined by gender and growth type. The data obtained can be used to optimize workflow for diagnosis, treatment planning, monitoring and outcome assessment of any given case. This is of special relevance in the virtual planning of orthognathic surgeries. Limitations of the study are population based and based on the cross-sectional study design. The dataset with only four subtypes of skulls, identified by gender and growth type, helps to serve as an individual guide for maxillofacial treatment in Eurasian patients, which is useful for orthodontic treatment, the 3D planning of orthognathic surgery and outcome assessment. We could show that the identification of statistically significantly different skull types is possible with our method. Therefore, it is reasonable to apply the technique to other ethnicities and malformations in further studies. Future research should provide insight into growth development in skull shape, as well as the detection of the extent of anatomical deviation in malformed patients.

## Figures and Tables

**Table 1:** List of the anatomical features used as landmarks.

No	Landmark	Abbreviation	Definition
1	Nasion	N	Intersection point of frontonasal and internasal suture
2	Frontoorbital suture right	FOS r	Intersection midpoint of processus zygomaticus and Os frontale
3	Frontoorbital suture left	FOS l	Intersection midpoint of proceccus zygomaticus and Os frontale
4	Inferior orbital edge right	IOE r	Midpoint in the curvature of the lateral orbita right
5	Inferior orbital edge left	IOE l	Midpoint in the curvature of the lateral orbita left
6	Incisura frontalis right	IF r	Medial border of the incisura frontalis right
7	Incisura frontalis left	IF l	Medial border of the incisura frontalis left
8	Spina nasalis anterior	SPA	The point on the tip of the Spina
9	First upper molar distobuccal root bone level right	UpMdbrbl r	Crestal edge of the upper right first molar distobuccal root
10	First upper molar distobuccal root bone level left	UpMdbrbl l	Crestal edge of the upper left first molar distobuccal root
11	Lower incisor bone level	LIbl	Crestal midpoint between lower first incisor roots
12	First lower molar distal root bone level right	LoMdbrbl r	Crestal edge of the lower right first molar distal root
13	First lower molar distal root bone level left	LoMdbrbl l	Crestal edge of the lower left first molar distal root
14	Condyle right	Co r	Most superior point on the midline of the condyle right
15	Condyle left	Co l	Most superior point on the midline of the condyle left
16	Pogonion	Po	Most anterior point of the mandibular symphysis
17	Posterior inferior mandibular point right	Pom r	Most caudal and most posterior point of the right mandibular
18	Posterior inferior mandibular point left	Pom l	Most caudal and most posterior point of the left mandibular

**Table 2:** List of distances determined from the landmarks in Table 1.

Abbr.	Distance
V1	Nasion (1)—Pogonion (16)
V2	Nasion (1)—SPA (8)
V3	SPA (8)—Pogonion (16)
V4	FOS r (2)—IOE r (4)
H1	FOS r (2)—FOS l (3)
H2	Co r (14)—Co l (15)
H3	UpMdbrbl r (9)—UpMdbrbl l (10)
Z1	IOE r (4)—Co r (14)

**Table 3:** List of proportions calculated from the measured distances in Table 2.

Abbr.	Proportion
P01	V1/H1
P02	V1/H3
P03	V1/H2
P04	V1/V3
P05	V1/V6
P06	V1/Z1
P07	V3–H1
P08	H1/H3

**Table 4:** Mean values of female and male proportions. The *p*-values of the *t*-test adjusted with Benjamini–Hochberg correction for multiple tests.

Proportion	Female	Male	Adj. *p*-Value
P01	1.08	1.115	0.031
P02	1.877	2.008	0.001
P03	1.04	1.082	0.015
P04	0.962	0.986	0.275
P05	4.851	5.293	0
P06	1.806	1.874	0.031
P07	1.816	1.785	0.344
P08	1.738	1.802	0.009

**Table 5:** Mean values of two clusters of male proportions. The *p*-values of the *t*-test adjusted with Benjamini—Hochberg correction for multiple tests.

Proportion	Cluster m1	Cluster m2	Adj. *p*-Value
P01	1.178	1.071	0
P02	2.146	1.912	0
P03	1.136	1.043	0
P04	0.94	1.019	0.005
P05	5.535	5.123	0.007
P06	1.977	1.802	0
P07	1.643	1.884	0
P08	1.823	1.786	0.197

**Table 6:** Mean values of two clusters of female proportions. The *p*-values of the *t*-test adjusted with Benjamini-Hochberg correction for multiple tests.

Proportion	Cluster f1	Cluster f2	Adj. *p*-Value
P01	1.164	1.034	0
P02	2.001	1.791	0
P03	1.092	1.003	0
P04	0.921	0.989	0.039
P05	4.979	4.763	0.147
P06	1.9	1.742	0
P07	1.67	1.917	0
P08	1.746	1.733	0.713

## Data Availability

The original landmark data are available from the corresponding author upon reasonable request.

## References

[1] Meyer U. (2021). Fundamentals of Craniofacial Malformations.

[2] Kerkfeld V., Schorn L., Depprich R., Lommen J., Wilkat M., Kübler N., Rana M., Meyer U. (2022). Simultaneous PSI-Based Orthognathic and PEEK Bone Augmentation Surgery Leads to Improved Symmetric Facial Appearance in Craniofacial Malformations. J. Pers. Med..

[3] Proffit W.R., Fields H.W., Sarver D.M. (2014). Contemporary Orthodontics e-Book.

[4] Broadbent B.H. (1931). A new X-ray technique and its application to orthodontia. Angle Orthod..

[5] Wang R.H., Ho C.T., Lin H.H., Lo L.J. (2020). Three-dimensional cephalometry for orthognathic planning: Normative data and analyses. J. Formos. Med. Assoc..

[6] Ho C.T., Denadai R., Lo L.J., Lin H.H. (2023). Average 3D Skeletofacial Model as a Template for Maxillomandibular Repositioning During Virtual Orthognathic Surgical Planning. Plast. Reconstr. Surg..

[7] Andriola F.D.O., Haas Junior O.L., Guijarro-Martínez R., Hernández-Alfaro F., Oliveira R.B.D., Pagnoncelli R.M., Swennen G.R. (2022). Computed tomography imaging superimposition protocols to assess outcomes in orthognathic surgery: A systematic review with comprehensive recommendations. Dentomaxillofac. Radiol..

[8] van Vlijmen O.J., Maal T.J., Berge S.J., Bronkhorst E.M., Katsaros C., Kuijpers-Jagtman A.M. (2009). A comparison between two-dimensional and three-dimensional cephalometry on frontal radiographs and on cone beam computed tomography scans of human skulls. Eur. J. Oral Sci..

[9] Yitschaky O., Redlich M., Abed Y., Faerman M., Casap N., Hiller N. (2011). Comparison of common hard tissue cephalometric measurements between computed tomography 3D reconstruction and conventional 2D cephalometric images. Angle Orthod..

[10] Gateno J., Xia J.J., Teichgraeber J.F. (2011). New 3-dimensional cephalometric analysis for orthognathic surgery. J. Oral Maxillofac. Surg..

[11] Devanna R. (2015). Two-dimensional to three-dimensional: A new three-dimensional cone-beam computed tomography cephalometric analysis. J. Orthod. Res..

[12] Jacobson A., Jacobson R.L. (2007). Radiographic Cephalometry: From Basics to 3-D Imaging.

[13] Swennen G.R., Schutyser F. (2006). Three-dimensional cephalometry: Spiral multi-slice vs cone-beam computed tomography. Am. J. Orthod. Dentofac. Orthop..

[14] Swennen G.R.J., Schutyser F.A.C., Hausamen J.E. (2005). Three-Dimensional Cephalometry: A Color Atlas and Manual.

[15] Bayome M., Park J.H., Kook Y.A. (2013). New three-dimensional cephalometric analyses among adults with a skeletal class I pattern and normal occlusion. Korean J. Orthod..

[16] Cheung L.K., Chan Y.M., Jayaratne Y.S., Lo J. (2011). Three-dimensional cephalometric norms of Chinese adults in Hong Kong with balanced facial profile. Oral Surg. Oral Med. Oral Pathol. Oral Radiol. Endodontol..

[17] Vahdettin L., Aksoy S., Oz U., Orhan K. (2016). Three-dimensional cephalometric norms of Turkish Cypriots using CBCT images reconstructed from a volumetric rendering program in vivo. Turk. J. Med. Sci..

[18] Gu Y., McNamara J.A., Sigler L.M., Baccetti T. (2011). Comparison of craniofacial characteristics of typical Chinese and Caucasian young adults. Eur. J. Orthod..

[19] Hwang H.S., Kim W.S., McNamara J.A. (2002). Ethnic differences in the soft tissue profile of Korean and European-American adults with normal occlusions and well-balanced faces. Angle Orthod..

[20] Adams G.L., Gansky S.A., Miller A.J., Harrell W.E., Hatcher D.C. (2004). Comparison between traditional 2-dimensional cephalometry and a 3-dimensional approach on human dry skulls. Am. J. Orthod. Dentofac. Orthop..

[21] Nalcaci R., Öztürk F., Sökücü O. (2010). A comparison of two-dimensional radiography and three-dimensional computed tomography in angular cephalometric measurements. Dentomaxillofac. Radiol..

[22] Lee S.H., Kil T.J., Park K.R., Kim B.C., Kim J.G., Piao Z., Corre P. (2014). Three-dimensional architectural and structural analysis—A transition in concept and design from Delaire’s cephalometric analysis. Int. J. Oral Maxillofac. Surg..

[23] Dryden I.L., Mardia K.V. (2016). Statistical Shape Analysis.

[24] Schlager S., Zheng G., Li S., Székely G. (2017). Morpho and R. Statistical Shape and Deformation Analysis.

[25] Celebi A.A., Tan E., Gelgor I.E., Colak T., Ayyildiz E. (2013). Comparison of soft tissue cephalometric norms between Turkish and European-American adults. Sci. World J..

[26] Mozzo P., Procacci C., Tacconi A., Martini P.T., Andreis I.A. (1998). A new volumetric CT machine for dental imaging based on the conebeamtechnique: Preliminary results. Eur. Radiol..

[27] Lopes P.M., Moreira C.R., Perrella A., Antunes J.L., Cavalcanti M.G. (2008). 3-D volume rendering maxillofacial analysis of angular measurements by multislice CT. Oral Surg. Oral Med. Oral Pathol. Oral Radiol. Endodontol..

[28] Farkas L.G., Tompson B., Phillips J.H., Katic M.J., Cornfoot M.L. (1999). Comparison of anthropometric and cephalometric measurements of the adult face. J. Craniofac. Surg..

[29] Oz U., Orhan K., Abe N. (2011). Comparison of linear and angular measurements using two-dimensional conventional methods and three-dimensional cone beam CT images reconstructed from a volumetric rendering program in vivo. Dentomaxillofac. Radiol..

[30] Ursi W.J., Trotman C.A., McNamara J.A., Behrents R.G. (1993). Sexual dimorphism in normal craniofacial growth. Angle Orthod..

[31] Humphrey L.T. (1998). Growth patterns in the modern human skeleton. Am. J. Phys. Anthropol..

[32] Rosas A., Bastir M. (2002). Thin-plate spline analysis of allometry and sexual dimorphism in the human craniofacial complex. Am. J. Phys. Anthropol..

[33] Bastir M. (2008). A systems-model for the morphological analysis of integration and modularity in human craniofacial evolution. J. Anthropol. Sci..

[34] Fearon J.A., Ditthakasem K., Herbert M., Kolar J. (2017). An appraisal of the cephalic index in sagittal craniosynostosis, and the unseen third dimension. Plast. Reconstr. Surg..

[35] Muralidhar N.V., Ranjan A., Jayashankar Rao J.S., Sreeshyla H.S., Nitin P. (2021). Cephalic index, facial index and dental parameters: A correlative study to evaluate their significance in facial reconstruction. J. Oral Maxillofac. Pathol..

[36] Bianchi A., Seidita F., Badiali G., Lusetti L., Saporosi C., Pironi M., Marchetti C., Crimi S. (2023). Is Beauty a Matter of Volume Distribution? Proposal of a New Aesthetic Three-Dimensional Guide in Orthognathic Surgery. J. Pers. Med..

[37] Baldini B., Cavagnetto D., Baselli G., Sforza C., Tartaglia G.M. (2022). Cephalometric measurements performed on CBCT and reconstructed lateral cephalograms: A cross-sectional study providing a quantitative approach of differences and bias. BMC Oral Health.

[38] Farronato M., Baselli G., Baldini B., Favia G., Tartaglia G.M. (2022). 3D Cephalometric Normality Range: Auto Contractive Maps (ACM) Analysis in Selected Caucasian Skeletal Class I Age Groups. Bioengineering.

